# Sclerostin Suppression Facilitates Uveal Melanoma Progression Through Activating Wnt/β-Catenin Signaling *Via* Binding to Membrane Receptors LRP5/LRP6

**DOI:** 10.3389/fonc.2022.898047

**Published:** 2022-06-17

**Authors:** Hanqing Wang, Sidi Zhao, Yang Liu, Fengyuan Sun, Xiaoming Huang, Tong Wu

**Affiliations:** ^1^ Department of Orbital Disease and Oculoplastic Surgery, Sichuan Eye Hospital, Aier Eye Hospital Group, Chengdu, China; ^2^ Department of Orbital Disease and Oculoplastic Surgery, Tianjin Medical University Eye Hospital, Tianjin, China; ^3^ Research and Development Department, Microsensor Labs, Chicago, IL, United States

**Keywords:** uveal melanoma, sclerostin, Wnt/β-catenin signaling, LRP5/LRP6, progression

## Abstract

**Objective:**

Uveal melanoma (UM) is the most frequent primary eye cancer in adults with a 50% mortality rate. Characterizing the fundamental signaling pathways that drive UM is of importance for the development of targeted therapy. This study aims to probe the impact of sclerostin (SOST) on malignant progression of UM and regulation of Wnt/β-catenin signaling.

**Methods:**

Epithelial-type (n=20) and spindle-type (n=16) UM tissues were collected for immunohistochemical staining of SOST, Wnt-1, and β-catenin expressions. SOST was silenced in three UM cell lines (primary spindle-type OCM-1 cells, metastatic epithelial Mum-2B cells, and metastatic spindle-type Mum-2C cells) through transfecting specific siRNA. RT-qPCR and Western blot were presented for examining the levels of SOST, and markers in Wnt/β-catenin signaling. Flow cytometry, MTT, EdU, transwell, and tube formation assays were conducted, respectively. By implanting BALB/c nude murine models *in situ*, the function of SOST on tumor growth was investigated, followed by immunofluorescence double staining of SOST and LRP5/6.

**Results:**

Low SOST expression as well as high Wnt-1 and β-catenin expressions were found in epithelial-type (high malignancy) than spindle-type (low malignancy) UM tissues. Silencing SOST activated the markers in Wnt/β-catenin signaling as well as accelerated cell cycle progression, migration, invasion, angiogenesis, and reduced apoptosis in UM cells. *In situ* tumor formation in murine eyes showed that SOST knockdown promoted tumor growth. Moreover, SOST interacted with LRP5/LRP6.

**Conclusion:**

SOST silencing may facilitate the malignant progression of UM cells through activating Wnt/β-catenin signaling. Mechanistically, SOST may exert this function by interacting with LRP5/LRP6 membrane receptors.

## Introduction

Uveal melanoma (UM) represents the most frequent primary intraocular tumor in adults as well as the second most frequent form of melanoma (1). It originates from the pigmented uveal tract, composed of choroid (~85%), ciliary body (5-8%), and iris (3-5%) (2, 3). In accordance with the WHO classification of histological types in 1980, UM is categorized as spindle cell type with low malignancy, epithelioid cell type with high malignancy, mixed cell type, and other types (4). Current therapeutic strategies include surgery, radiotherapy, and chemotherapy as well as targeted therapy, and the local control rate of UM has reached as high as 96.4% following active treatment (5). Nevertheless, approximately 50% of cases will develop metastasis within 15 years following the initial diagnosis (6, 7). Regrettably, there is still no effective treatment against metastasis (8). UM possesses an extremely high predilection to liver metastasis, and exceeding 90% of metastatic patients have hepatic lesions (9). At present, the prognosis of tumor invasion and metastasis cannot be accurately and objectively predicted in clinical practice, and there are not many corresponding effective treatment methods, which are the main factors affecting the curative effect and patient survival rate of UM (10). Therefore, it is necessary to conduct in-depth research on the mechanism of tumorigenesis and tumor metastasis, and to clarify the molecular biological characteristics of this disease.

Wnt/β-catenin signaling exerts a crucial role in tumor progression (11), and blockage of this signaling may suppress growth, migration, and invasion of UM cells (12–14). Sclerostin (SOST) secreted by osteocytes is a negative regulator of bone formation, and is an antagonist of the DAN/Cerberus protein family of BMP (15). Evidence suggests that SOST acts as a potential antagonist of Wnt/β-catenin pathway and inactivated SOST leads to the hyper-activation of this signaling (16). Several studies have reported that SOST-mediated Wnt/β-catenin pathway participates in retinoblastoma progression (17) and osteosarcoma (18). Evidence suggests that SOST can antagonize Wnt signaling in human cells *via* binding to the extracellular domain of LRP5/6 Wnt co-receptors as well as destroying Wnt-triggered Frizzled-LRP complexes (19). Nevertheless, the roles of SOST-triggered Wnt/β-catenin pathway in UM development as well as its clinical implication remain unclear. Hence, our study was conducted for probing the impact of SOST on malignant progression of UM and regulation of Wnt/β-catenin signaling.

## Materials and Methods

### Patients and Specimens

Twenty epithelial-type UM tissue specimens and 16 spindle-type UM tissue specimens were retrieved from UM patients who underwent enucleation in Tianjin Medical University Eye Hospital from October 2015 to April 2018. All patients were confirmed by pathological examination after operation, who were classified according to the 1980 WHO classification of histological types. The age range was 24-67 years. The inclusion criteria were as follows (1): the pathological diagnosis was primary UM; (2) the submitted tissue sections were relatively complete; (3) the patient had no other types of cancer; and (4) the patient had not received radio- or chemotherapy. This study was approved by the Ethics Committee of Tianjin Medical University Eye Hospital (2017KY(L)-53). All patients provided written informed consent.

### Immunohistochemistry

The patients’ eye tumor specimens were fixed in neutral formalin and embedded in paraffin, followed by sectioning, deparaffinization, antigen retrieval, blocking, antibody incubation, DAB (diaminobenzidine) staining, hematoxylin staining, back blue, and dehydration coverslips. Primary antibodies were anti-rabbit (Proteintech, China), and secondary antibodies were goat anti-rabbit IgG (H+L) (Jackson Immuno Research Inc., US). As previously described, the staining scoring was calculated according to the following formula: H-score = staining intensity * number of positive cells/total number of cells (20). Three random fields were chosen, and the staining intensity was manually evaluated: 0 point for no yellow precipitate, 1 point for light yellow precipitate, 2 points for brownish-yellow precipitate, and 3 points for dark brown-yellow precipitate; positive cell counts were manually counted using Image J software.

### Cell Culture

Human retinal epithelial cell line (APRE-19), human umbilical vein endothelial cells (HUVEC) as well as three human UM cell lines were acquired from the Chinese Academy of Sciences (Shanghai, China), including primary spindle-type OCM-1 cells, metastatic epithelial Mum-2B cells, and metastatic spindle-type Mum-2C cells. All cells were cultured in an incubator (Thermo Fisher Scientific, US) with 37°C, 5% CO_2_. Subculture was carried out when the cells adhered and reached about 80% confluence. After discarding the medium, they were washed with Dulbecco’s Phosphate-Buffered Saline (DPBS; Gibco, US), and digested with 1 mL of 0.25% trypsin-EDTA (Gibco, US). Thereafter, Dulbecco’s modified eagle medium (DMEM) containing 100 U/mL penicillin, 100 μg/mL streptomycin, and 10% fetal bovine serum (FBS; Gibco, US) was used to terminate the digestion. The cells were centrifuged at 1000 rpm and resuspended in DMEM for future experiments.

### Western Blot

UM cells were lysed by RIPA cell lysate, and protein concentrations were measured utilizing BCA Protein Assay Kit (Thermo Scientific, US). There were 20 μg proteins separated by 10% SDS-PAGE electrophoresis as well as transferred onto polyvinylidene fluoride membrane. The membrane was sealed with 5% nonfat dry milk and then incubated with primary antibodies against SOST (1:1000; 21933-1-AP; Proteintech, China), Wnt-1 (1:1000; 27935-1-AP), β-catenin (1:5000; 51067-2-AP), cyclin-D1 (1:2000; 26939-1-AP), MMP2 (1:500; 10373-2-AP), and MMP9 (1:500; 10375-2-AP) as well as β-Tubulin (1:1000; 10094-1-AP) at 4°C overnight. The next day, the membrane was incubated with goat anti-rabbit secondary antibody (Cell Signaling Technology, US) for 1 h at room temperature. Exposure was performed using Amersham ECL prime chemiluminescent fluid (GE Healthcare, US) in a dark room. The gray value was determined with ImageJ software.

### Cell Transfection

The design and synthesis of the sequences of siRNA targeting human SOST (gene ID: 50964) and negative control siRNA (si-NC) were achieved by Shanghai Sangon Bioengineering Co., Ltd., (China). The sequence of SOST siRNA (si-SOST) was 5′-GGCGTTCAAGATGATGCCACGGAA-3′. OCM-1, Mum-2B, and Mum-2C cell lines were randomly divided into three groups: blank group (no transfection), si-NC group (transfection with si-NC plasmid), and si-SOST group (transfection with si-SOST plasmid). The full length SOST cDNA was synthesized and sub-cloned into the pcDNA3.1 vector to construct pcDNA3.1-SOST overexpression (OE-SOST) plasmid. OCM-1, Mum-2B, and Mum-2C cell lines were randomly divided into two groups: empty vector group and OE-SOST group. These cells were trypsinized at 0.25% as well as centrifuged at 1000 rpm lasting 5 min. Then, they were resuspended in medium and counted using an automated cell counter (Invitrogen, US), and 4×10^5^ cells/well were seeded onto a six-well culture plate. When the cell confluency reached 50-80%, the complete medium was replaced with serum- and penicillin-free medium. Four hours later, using Lipofectamine 2000 (Invitrogen, US), plasmids encoding target sequences or siRNAs were transfected into the UM cell line. Twenty-four hours after transfections, the complete medium was exchanged for subculture. During this period, cellular morphology was investigated under a microscope (Olympus, Tokyo, Japan).

### Real-Time Quantitative Polymerase Chain Reaction (RT-qPCR)

Total RNAs of UM cells were extracted utilizing Trizol kit (Invitrogen, US). The extracted RNAs were utilized for reverse transcription. Cellular cDNA synthesis was conducted according to the instructions of the reverse transcription kit (Transgen, Beijing, China). The PCR amplification reaction was carried out according to the instructions of RT-qPCR kit (Transgen, Beijing, China). **Table 1** lists the primer sequences that were synthesized by Shanghai Sangon Bioengineering Co., Ltd. (China). The reaction condition contained pre-denaturation at 94°C lasting 4 min, denaturation at 94°C lasting 30 sec, annealing at 56°C lasting 30 sec, and extending at 72°C lasting 30 sec, in total 45 cycles. The reaction system contained 10 μl Premix Ex Taq or SYBR Green Mix, 1 μl forward primer, 1 μl reverse primer, 1 μl cDNA template, and ddH_2_O supplemented to 20 μl. Glyceraldehyde phosphate dehydrogenase (GAPDH) acted as an internal reference gene, and the Ct values ​​(threshold of amplification curves) were examined with the control group set to 1. The iQ5 Real-Time PCR Amplifier was purchased from the Bio-Rad company (US). Relative expression values ​​were calculated by the 2^-ΔΔCt^ method.

**Table 1:** The primer sequence utilized for RT-qPCR.

Gene name	Primer sequence (5’-3’)
SOST	Forward: TGAGACCAAAGACGTGTCCG Reverse: CTTGAGCTCCACTGGTTGT
WNT1	Forward: CGATGGTGGGGTATTGTGAAC Reverse: CCGGATTTTGGCGTATCAGAC
CTNNB1	Forward: ATGATGGTCTGCCAAGTGGG Reverse: GGCCATCTCTGCTTCTTGGT
CCND1	Forward: GCTGCGAAGTGGAAACCATC Reverse: CCTCCTTCTGCACACATTTGAA
MMP2	Forward: TACAGGATCATTGGCTACACACC Reverse: GGTCATCGCTCCAGACT
MMP9	Forward: TGTACCGCTATGGTTACACTCG Reverse: GGCAGGGACAGTTGCTTCT
GAPDH	Forward: GGAGCGAGATCCCTCCAAAAT Reverse: GGCTGTTGTCATACTTCTCATGG

### Cell Cycle Detection by Flow Cytometry

UM cells were collected and digested with trypsin, followed by centrifugation at 1000 rpm for 5 min. After rinsing the cells with sterile PBS, 1 ml of pre-cooled 70% ethanol was utilized to resuspend the cells as well as fixed overnight at 4°C. They were then rinsed with PBS to remove the ethanol solution, followed by incubation with 500 μl of PI staining solution at room temperature lasting 15 min in the dark. The cell cycle was detected by flow cytometry.

### Apoptosis Detection *via* Flow Cytometry

UM cells were trypsinized, followed by centrifugation at 1000 rpm lasting 5 min. After rinsing the cells utilizing pre-cooled sterile PBS at 4°C, the cellular concentrations were adjusted to 5×10^4^/mL. Then, 10 μl of Annexin V-FITC and 10 μl of PI were added to 200 μl of the cell suspension and incubated for 10 min at room temperature in the dark, followed by the addition of 500 μl PBS. Apoptotic levels were examined by flow cytometry (BD, US) as well as analyzed with FlowJo flow cytometry software.

### Methyl Thiazolyl Tetrazolium (MTT)

Following adjustment of the cellular concentration to 1×10^4^/mL, the cells were seeded in 96-well plates, with six duplicate wells for each group. The edge wells were filled with 0.01 mol/L sterile PBS. After 24 h at constant temperature, the medium in the wells was aspirated and discarded, and 90 μL sterile PBS supplemented with 10 μL 0.5% MTT (Sigma-Aldrich, US) were added. After 4 h, the culture was terminated. Thereafter, 100 μl of dimethyl sulfoxide (DMSO; Sigma-Aldrich, US) was added to each well. The plate was shaken well for 10 min, and the absorbance (optical density, OD) value at 490 nm wavelength of each well was measured with a microplate reader (Promega, US).

### EdU Staining

UM cells were seeded in 24-well plates (1 mL/well) containing 1 cm × 1 cm sterile glass slides. The plates were pre-incubated for 24 h at 37°C in a 5% CO_2_ incubator. After transfection, 20 μM EdU working solution pre-warmed at 37°C was added to a 24-well plate. The cells continued to incubate for 2 h. After removing the culture medium, the cells were fixed with 1 ml of 4% paraformaldehyde for 15 min at room temperature. After washing, the cells were incubated with 1 ml of 0.3% TritonX-100 in PBS per well lasting 10 min at room temperature, followed by 0.5 ml Click addictive reaction lasting 30 min in the dark. DAPI (1000×) was diluted 1:1000 in PBS. After removing the washing solution, they were incubated with 200 μl of 1× DAPI solution at room temperature lasting 5 min in the dark. Finally, fluorescence detection was performed.

### Transwell Assays

For invasion assay, Matrigel was diluted 8:1 with serum-free DMEM. Then 100 μL diluted Matrigel was added to the upper chamber of the transwell, and the whole process was performed on ice to prevent it from solidifying. The residual liquid was aspirated from the culture plate. UM cells were trypsinized, resuspended, and concentration adjusted. The upper chamber of a 24-well plate transwell covered with Matrigel was evenly seeded 1×10^5^ cells/well as well as maintained by adding serum-free DMEM. Meanwhile, 600 μl of complete medium with 10% FBS was added to the lower chamber. Following 24 h, the chamber was removed, rinsed twice with PBS, and then gently wiped with a cotton swab to remove unpenetrated cells on the upper layer of the chamber membrane. The cells were fixed with 4% paraformaldehyde for 30 min in a 24-well plate, stained with crystal violet staining solution lasting 15 min. The cells invaded through Matrigel were observed under an inverted microscope. Three areas were randomly selected to count the number of invasive cells through Matrigel. The experimental procedure for the migration assay was the same as for the invasion assay, except that Matrigel was not added.

### Tube Formation Assay

UM cells transfected with si-NC or si-SOST were co-cultured with HUVEC, and the tube formation ability was detected. Briefly, Matrigel was removed from -20°C and placed in a 4°C refrigerator overnight. The next day, 50 μl Matrigel was added to pre-cooled 96-well plates, and maintained in a 37°C incubator for 45 min. The co-cultured HUVEC was routinely digested, resuspended in cell medium containing 10% FBS. Thereafter, they were seeded into 96-well plates (1×10^4^ cells/well) as well as incubated at 37°C. The tube formation was investigated under an inverted microscope.

### Animal Experiment

The primer of SOST was designed by querying the CDS sequence on the NCBI website as well as the Thermo fisher website, with a primer sequence of 5’- AAAAGCAGGCGTTCAAGAATGATGCTTGGATCCAAGCATCATTCTTGAACGCCTGC-3’. The primer was synthesized by Shanghai Sangon Bioengineering Co., Ltd. (China). With pENTRTM H1/TO as vector, shRNA SOST (sh-SOST) or negative control (sh-NC) plasmids were constructed by enzyme digestion, ligation, transformation, and other operations, and then the plasmid identification was carried out. There were 293 T cells used to package the target plasmid, and when the density of OCM-1 cells reached 40%-50%, a stable transfection cell line was constructed after screening.

Six-week-old BALB/c nude female mice (14 ± 2 g) were purchased from Vital River Company (Beijing, China). All experiments gained the approval of the Laboratory Animal Care and Use Committee of Tianjin Medical University Eye Hospital (TJYY2020122054). All animals were separated into four groups (3 mice/group), containing a control group, model group, model + sh-NC group, and model + sh-SOST group. Normal OCM-1 cells or OCM-1 cells stably transfected with sh-NC or sh-SOST were trypsinized, centrifuged as well as suspended in Hanks’ balanced salt at 1×10^7^/mL. The abdominal skin of the mice was disinfected with iodophor, and the mice were anesthetized by intraperitoneal injection of 0.5% pentobarbital sodium. The right eye of the mice was instilled with proparacaine for ocular surface anesthesia, and the left eye was coated with ophthalmic gel to prevent keratitis. Under the operating microscope, a 30G needle was pointed at the oblique angle to create a tunnel *via* the sclera, and to penetrate the choroid without retina. There were 34G blunt needles attached to 10 μL Hamilton syringes inserted into the subretinal space with the bevel toward the inner eye. The mice in the model group, model + sh-NC group, and model + sh-SOST group were separately injected by 5 μL (5×10^4^ cells) of the cell suspension of normal OCM-1 cells or OCM-1 cells stably transfected by sh-NC or sh-SOST. For the control group, the mice did not receive any treatment. After the injection, a white bump was found in the fundus of the mouse, indicating that the injection was successful. Tobramycin ophthalmic ointment was then applied to the eye to reduce the risk of infection. After successful injection, the state of the mice was observed and weighed every other week, and the time of tumor formation in the eyes of the mice was recorded. After six weeks, all mice were euthanized, and their eyeballs were enucleated. The length (L) as well as width (W) of the murine eyeballs were measured utilizing a vernier caliper. The tumor volume (V) was measured in accordance with the following formula: V = π×L×W^2^×1/6.

### TUNEL Staining

A TUNEL kit was purchased from the Roche Company (Switzerland). The tissue slides were soaked in xylene for 10 min to dewax. For hydration, the sections were soak in graded ethanol (100%, 95%, 85%, 70%, 50%) for 30 sec, respectively. After washing twice with 1×PBS for 5 min each, 20 μg/ml proteinase K was dropwise added. After 15 min at 37°C, the sections were washed 3 times with 1×PBS. The labeling solution was mixed with buffer evenly at a volume ratio of 1:9. Thereafter, 100 μL TUNEL reaction solution was dropwise added. The reaction area with a cover glass was covered, and reacted at 37°C in a humid, dark environment for 60 min. After washing three times with 1×PBS, 50 μL of DAPI buffer (0.1 mg/mL; Sigma, US) was added to each well, and incubated in the dark at room temperature for 5 min. After washing 3 times with 1×PBS, images were photographed under a fluorescence microscope.

### Immunofluorescence

Paraffin sections of murine *in situ* UM tumors in eyeball were deparaffinized, antigen retrieved, blocked with 5% BSA, incubated with primary and secondary antibodies, and finally stained with DAPI (Sigma, US) and mounted with resin. Primary antibodies included SOST, LRP5, and LRP6 antibodies (Affinipure, Jackson, US), and fluorescent-labeled secondary antibodies included Alex Fluor 488 and Alex Fluor 610 goat anti-rabbit antibodies (Affinipure, Jackson, US). The sections were then observed under a fluorescence microscope and three fields of view were randomly selected to be photographed. The nuclei stained with DAPI were blue under the excitation of ultraviolet, and the positive expression was fluorescein-labeled green or red.

### Statistical Analysis

All statistical analysis was carried out utilizing GraphPad Prism software (version 8.0.1, GraphPad Software, US). Student’s t-test was implemented for comparisons between two groups, and one-way analysis of variance (ANOVA) was implemented for comparisons between multiple groups. Pearson correlation test was used for correlation analysis. P<0.05 was considered statistically significant.

## Results

### Down-Regulated SOST and Up-Regulated Wnt-1 and β-Catenin in Epithelial Type Than Spindle Type UM Tissues

**Figure 1:**
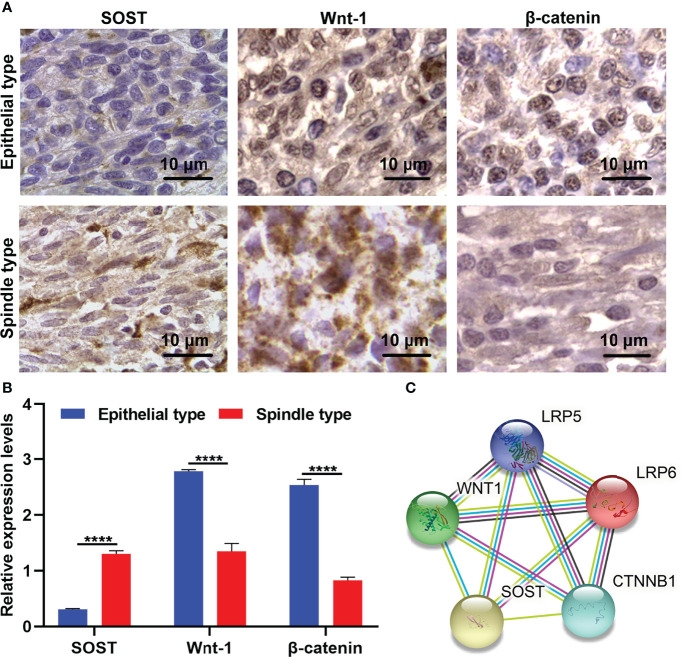
Abnormal expressions of SOST and Wnt-1 as well as β-catenin in spindle type than epithelial type UM tissues. **(A)** Representative immunohistochemistry images for SOST and Wnt-1 as well as β-catenin in human spindle type and epithelial type UM tissues. Magnification, ×100. Scale bar, 10 μm. **(B)** Immunohistochemistry staining scoring of SOST and Wnt-1 as well as β-catenin levels in human spindle type (n=16) and epithelial type (n=20) UM specimens. **(C)** The protein-protein interactions of SOST, Wnt-1, β-catenin, LRP5, and LRP6. ****p<0.0001.

Our study analyzed the expressions of SOST and Wnt-1 as well as β-catenin in 20 cases of epithelial type UM tissues with a high degree of malignancy and 16 cases of spindle type UM with a low degree of malignancy *via* immunohistochemistry. The results showed that SOST expression was lower as well as Wnt1 and β-catenin expressions were higher in epithelial type in comparison to spindle type UM tissues (**Figures 1A, B**). This indicated that low SOST expression as well as activation of Wnt/β-catenin signaling were linked to malignant progression of UM. We also calculated the correlation between SOST and Wnt1 and β-catenin in UM. Our results showed that SOST was negatively correlated to Wnt1 (p=0.0003 and r=-0.9869) and β-catenin (p<0.0001 and r=-0.9953). The STRING online database also confirmed their protein-protein interactions (**Figure 1C**).

### SOST Knockdown Activates Wnt/β-Catenin Signaling in UM Cells

**Figure 2:**
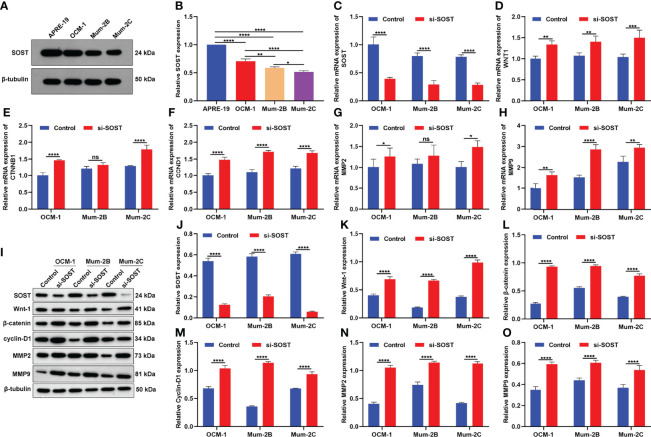
SOST knockdown activates Wnt/β-catenin signaling in UM cells. **(A**, **B)** Western blot of SOST expression in APRE-19 cells as well as three human UM cell lines OCM-1, Mum-2B, and Mum-2C. **(C–H)** RT-qPCR of the expressions of WNT1, CTNNB1, CCND1, MMP2, and MMP9 mRNAs in si-SOST-transfected OCM-1, Mum-2B, and Mum-2C cells and controls. **(I-O)** Western blot of SOST, Wnt-1, β-catenin, Cyclin-D1, and MMP2 as well as MMP9 levels in si-SOST-transfected OCM-1, Mum-2B, and Mum-2C cells and controls. Ns, no significance; *p<0.05; **p<0.01; ***p<0.001; ****p<0.0001.

SOST expression was further examined in human retinal epithelial cell line (APRE-19) and three human UM cell lines containing primary spindle-type OCM-1 cells, metastatic epithelial Mum-2B cells, and metastatic spindle-type Mum-2C cells. As shown in **Figures 2A, B**, SOST expression was lower in three human UM cell lines in comparison to APRE-19 cells. Compared with OCM-1 cells, lower SOST expression was found in Mum-2B and Mum-2C cells. Meanwhile, SOST expression was lower in Mum-2C cells than Mum-2B cells. This indicated the down-regulation of SOST in metastatic cells. To silence the expression of SOST, siRNA against SOST was transfected into OCM-1, Mum-2B, and Mum-2C cells. In **Figure 2C**, 48 h after transfection, the expression of SOST mRNA was successfully decreased in three human UM cell lines. Further analysis showed that the expressions of WNT1, CTNNB1, CCND1, MMP2, and MMP9 mRNAs were decreased in si-SOST-transfected OCM-1, Mum-2B, and Mum-2C cells than controls (**Figures 2D–H**). As expected, SOST protein was reduced following si-SOST transfection than control in three human UM cell lines (**Figures 2I, J**). Additionally, Wnt-1, β-catenin, Cyclin-D1, MMP2, and MMP9 proteins presented higher levels when SOST expression was knocked out (**Figures 2K–O**). Collectively, silenced SOST could activate the Wnt/β-catenin pathway in UM cells.

### Low SOST Expression Accelerates Cell Cycle Progression As Well As Reduces Apoptosis in UM Cells

**Figure 3:**
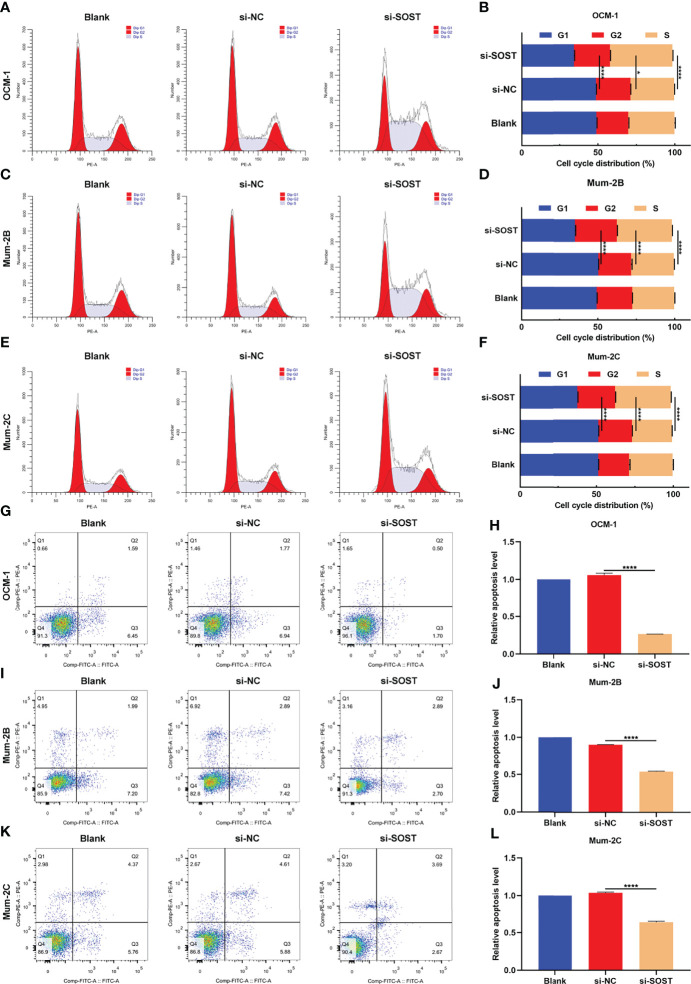
Low SOST expression accelerates cell cycle progression and reduces apoptosis in UM cells. **(A–F)** Cell cycle distribution of normal, si-NC-, or si-SOST-transfected OCM-1 and Mum-2B as well as Mum-2C cells. **(G–L)** Apoptotic levels of normal, si-NC-, or si-SOST-transfected OCM-1, Mum-2B, and Mum-2C cells. *p<0.05; ****p<0.0001.

Flow cytometry showed that after si-SOST transfection, the proportion of G1 phase was lower as well as the proportions of S phase and G2 phase were higher in three cell lines OCM-1, Mum-2B, and Mum-2C compared with that of the si-NC group, indicating that silencing SOST expression was capable of increasing the proportion of UM cells in interphase (**Figures 3A–F**). Additionally, the apoptotic levels of si-SOST-transfected OCM-1, Mum-2B, and Mum-2C cells were decreased in comparison to that of the si-NC group (**Figures 3G–L**). Altogether, SOST knockdown enabled us to accelerate cell cycle progression as well as decrease apoptosis in UM cells.

### SOST Knockdown Strengthens Proliferative Capacities of UM Cells

**Figure 4:**
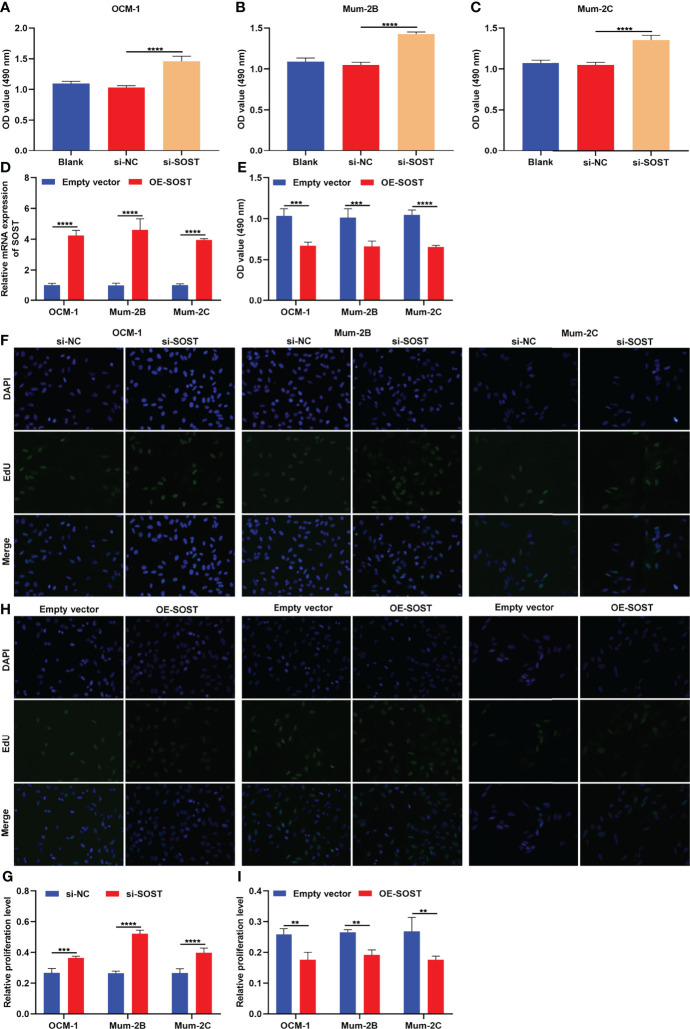
SOST knockdown strengthens proliferative capacities of UM cells. **(A–C)** CCK-8 for the cell viability of normal, si-NC-, or si-SOST-transfected OCM-1, Mum-2B, and Mum-2C cells. **(D)** RT-qPCR for the expression of SOST in empty vector- or OE-SOST-transfected OCM-1, Mum-2B, and Mum-2C cells. **(E)** CCK-8 for the cell viability of empty vector- or OE-SOST-transfected OCM-1, Mum-2B, and Mum-2C cells. **(F**, **G)** EdU staining for the proliferation of normal, si-NC-, or si-SOST-transfected OCM-1, Mum-2B, and Mum-2C cells. **(H**, **I)** EdU staining for the proliferation of empty vector- or OE-SOST-transfected OCM-1, Mum-2B, and Mum-2C cells. Magnification, ×200. Scale bar, 10 μm. **p<0.01; ***p<0.001; ****p<0.0001.

Through CCK-8 as well as EdU staining assays, this research evaluated the effects of SOST on proliferation of UM cells. As depicted in **Figures 4A–C**, the cell viability of si-SOST-transfected OCM-1, Mum-2B, and Mum-2C cells was elevated in comparison to that of the si-NC group. To overexpress SOST, UM cells were transfected with OE-SOST plasmid. RT-qPCR confirmed that SOST expression was significantly up-regulated in UM cells (**Figure 4D**). Lower cell viability was found in SOST-overexpressed OCM-1, Mum-2B, and Mum-2C cells (**Figure 4E**). Moreover, proliferation levels of three human UM cell lines were enhanced by si-SOST transfection (**Figures 4F, G**) as well as weakened by SOST overexpression (**Figures 4H, I**). Thus, reduced expression of SOST enabled us to strengthen proliferative capacities of UM cells.

### Silencing SOST Facilitates Migration, Invasion, and Angiogenesis of UM Cells

**Figure 5:**
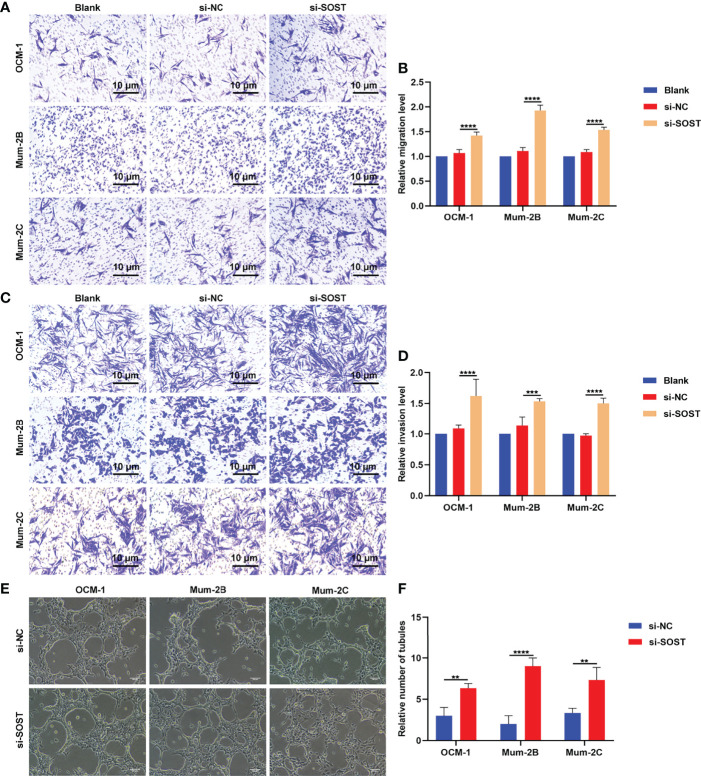
Silencing SOST facilitates migration and invasion as well as angiogenesis of UM cells. **(A**, **B)** Transwell experiments for the migration of normal, si-NC-, or si-SOST-transfected OCM-1, Mum-2B, and Mum-2C cells. Magnification, ×40. Scale bar, 10 μm. **(C**, **D)** Transwell experiments for the invasion of normal, si-NC-, or si-SOST-transfected OCM-1, Mum-2B, and Mum-2C cells. Magnification, ×40. Scale bar, 10 μm. **(E**, **F)** Tube formation experiment of HUVEC co-cultured with normal, si-NC-, or si-SOST-transfected OCM-1, Mum-2B, and Mum-2C cells. Magnification, ×40. Scale bar, 200 μm. **p<0.01; ***p<0.001; ****p<0.0001.

Transwell experiment results showed that compared with the si-NC group, the number of OCM-1, Mum-2B, and Mum-2C cells that penetrated the upper chamber of transwell in the si-SOST group was elevated in the si-SOST group (**Figures 5A, B**). This indicated the increase in migration capacities of UM cells. In **Figures 5C, D**, the number of three human UM cell lines invaded through Matrigel was increased in the si-SOST group in comparison to the si-NC group, indicating the enhancement in invasion capacities of UM cells. Following co-culture with si-SOST-transfected OCM-1, Mum-2B, and Mum-2C cells, the tube formation number of HUVEC was increased (**Figures 5E, F**). Altogether, SOST knockdown could enhance migration and invasion as well as angiogenesis of UM cells.

### SOST Suppression Strengthens Tumor Growth in Murine Models

**Figure 6:**
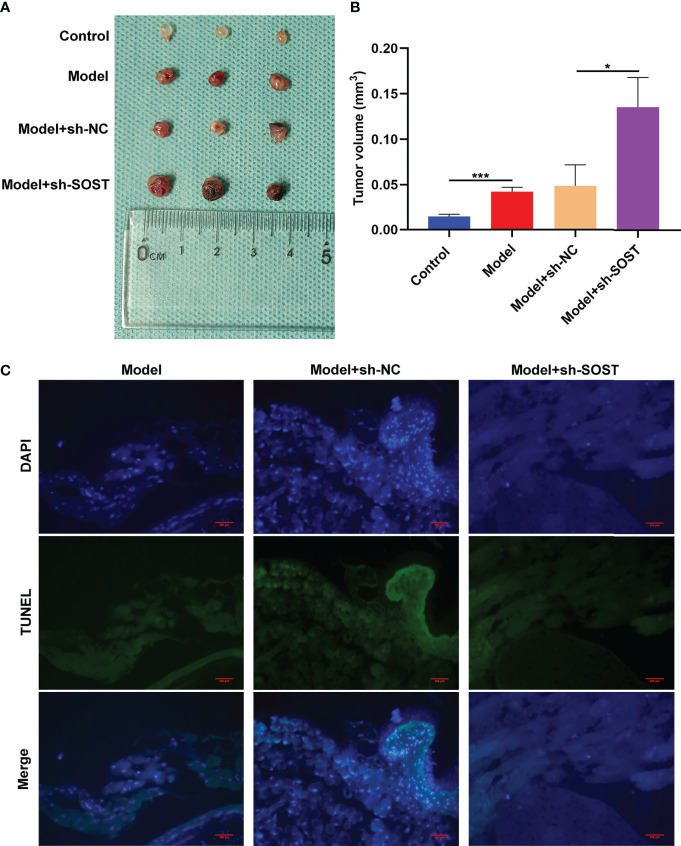
SOST suppression strengthens tumor growth in murine models. **(A)** Representative images of tumors in eyeballs from control mice or those injected with normal, sh-NC-, or sh-SOST-transfected OCM-1 cells after six weeks. **(B)** Measurement of tumor volume of each group. *p<0.01; ***p<0.001. **(C)** TUNEL staining of tumor sections from mice injected with normal, sh-NC-, or sh-SOST-transfected OCM-1 cells after six weeks. Magnification, ×40. Scale bar, 200 μm.

To further verify the impact of SOST on UM, we constructed a stable SOST knockdown expression OCM-1 cells as well as ocular orthotopic tumor models in nude mice. Following six weeks, we removed the eyeballs and measured the tumor volume. In comparison to the control group, the tumor volume of the model group was increased. Meanwhile, higher tumor volume was found in the model + sh-SOST group than the model + sh-NC group (**Figures 6A, B**), indicating that SOST suppression accelerated tumor growth. TUNEL staining also demonstrated that proliferative capacity of tumor cells in the model + sh-SOST group was heightened in comparison to that of the model + sh-NC group (**Figure 6C**).

### SOST Suppression Activates Wnt/β-Catenin Signaling in Ocular Orthotopic Tumor Models

**Figure 7:**
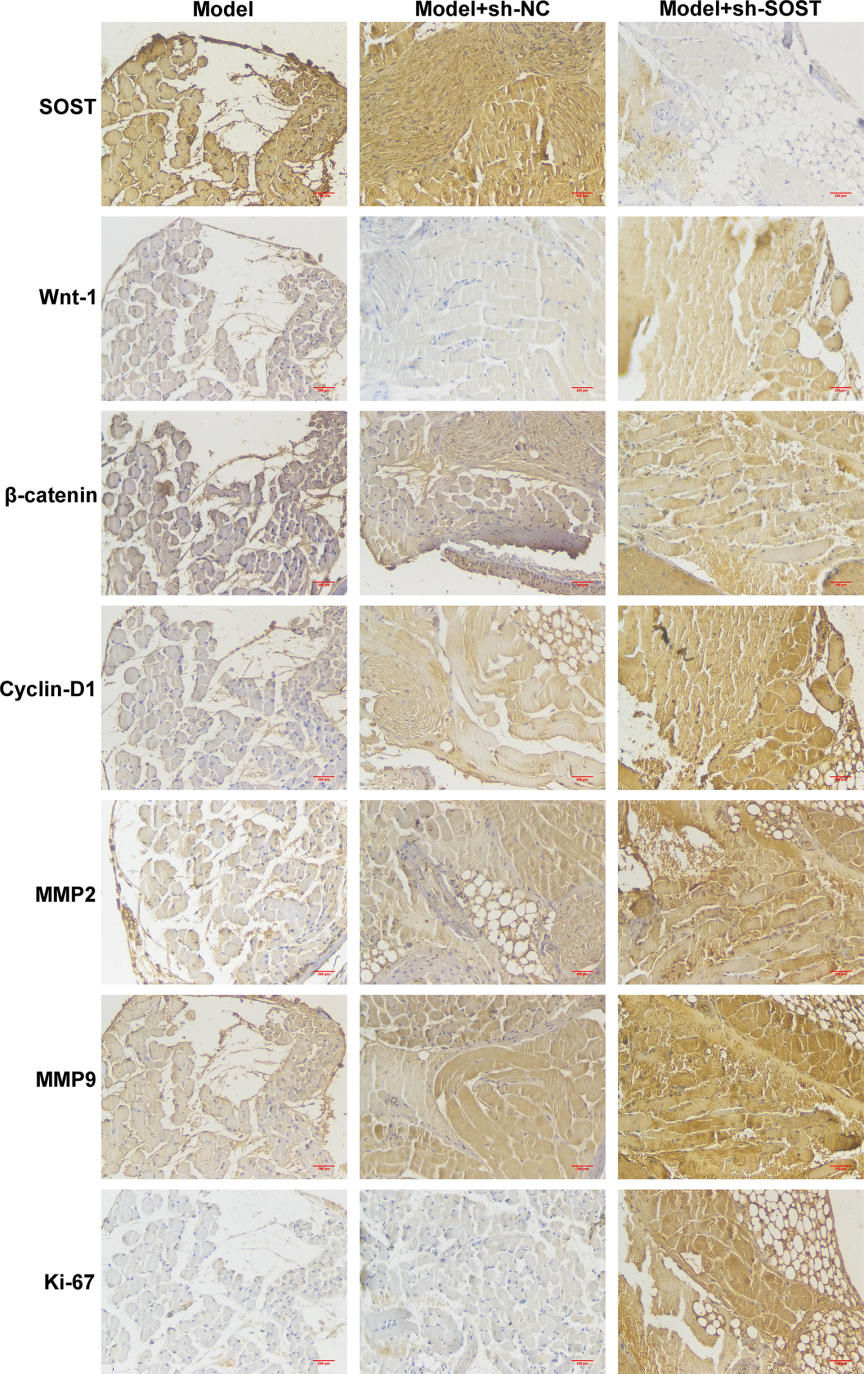
SOST suppression activates Wnt/β-catenin pathway in ocular orthotopic tumor models. Immunohistochemistry staining for SOST, Wnt-1, β-catenin, cyclin-D1, MMP2, and MMP9 as well as Ki-67 in tumor sections from mice injected with normal, sh-NC-, or sh-SOST-transfected OCM-1 cells after six weeks. Magnification, ×40. Scale bar, 200 μm.

Immunohistochemistry demonstrated that SOST expression was lower in tumor cells of the model + sh-SOST group in comparison to that of the model + sh-NC group (**Figure 7**). Additionally, Wnt-1, β-catenin, cyclin-D1, MMP2, and MMP9 as well as Ki-67 levels were higher in tumor cells of the model + sh-SOST group than that of the model + sh-NC group, demonstrating that SOST suppression was capable of activating Wnt/β-catenin signaling in ocular orthotopic tumor models.

### Interaction Between SOST and LRP5/6 in UM Cells

**Figure 8:**
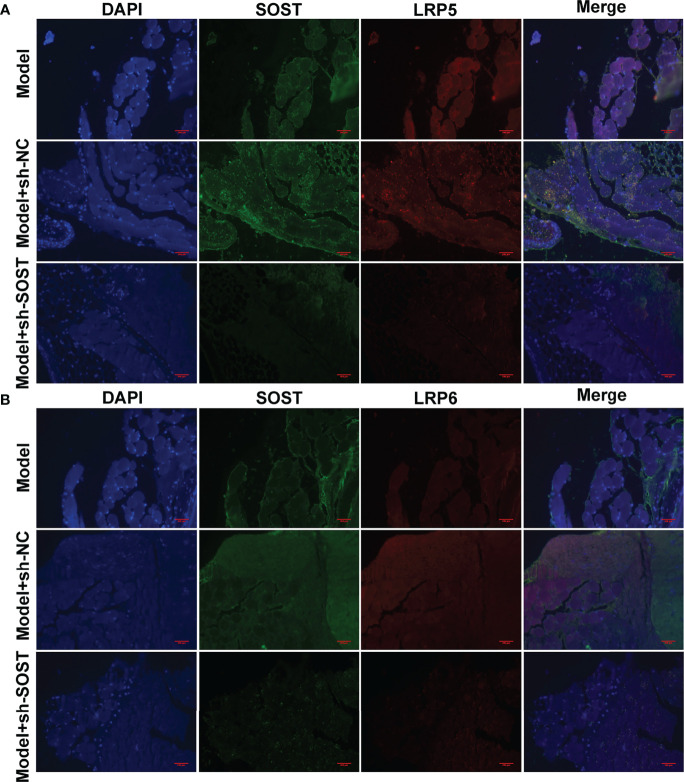
Interaction between SOST and LRP5/6 in UM cells. **(A**, **B)** Immunofluorescence double staining of SOST and LRP5/6 in tumor sections from mice injected with normal, sh-NC-, or sh-SOST-transfected OCM-1 cells after six weeks. Magnification, ×40. Scale bar, 200 μm.

Further analysis showed that LRP5 and LRP6 membrane receptors presented lower expressions in tumor cells of the model + sh-SOST group than that of the model + sh-NC group, demonstrating that SOST suppression could decrease the expressions of LRP5 and LRP6 (**Figures 8A, B**). Also, SOST co-localized with LRP5/6 in UM cells, indicating that SOST was interacted with LRP5/6.

## Discussion

UM is an aggressive malignancy that originates from ocular melanocytes, which is an incurable melanoma and the mortality rate is as high as 50% (21, 22). The experimental results from this study demonstrated that SOST silencing could heighten the proliferation, migration, and invasion as well as angiogenesis of human UM cells *via* activating Wnt/β-catenin signaling *via* binding to membrane receptors LRP5/LRP6.

Low SOST expression as well as high Wnt-1 and β-catenin expressions were found in epithelial-type (high malignancy; n=20) in comparison to spindle-type (low malignancy; n=16) UM tissues. Silencing SOST activated Wnt/β-catenin signaling in three human UM cell lines. Moreover, its knockdown accelerated cell cycle reduced apoptosis as well as facilitated migration, invasion, and angiogenesis of UM cells. SOST is primarily expressed in osteocytes, as a negative regulator of bone formation (23, 24). SOST deficiency facilitates bone formation *via* Wnt/β-catenin signaling pathway as well as compensates particle-triggered osteolysis (25). Accumulated evidence has demonstrated that SOST mediates cancer progression. Hudson et al. found that SOST participates in the early onset of bone metastasis of prostate cancer, which can inhibit the invasion of prostate cancer cells as well as subsequent bone metastasis (26). SOST deficiency triggers proliferative and invasive capacities and decreases apoptotic levels of human retinoblastoma cells (17) and osteosarcoma cells (18) *via* activation of Wnt/β-catenin signaling. Hesse et al. reported that suppressing SOST is capable of alleviating breast cancer-triggered bone metastasis and muscle weakness (27). In postmenopausal Romanian women with osteoporosis, there is a close association between LRP5 and SOST polymorphisms (28). Evidence suggests that LRP5/6 co-deletion in bone markedly reduces the skeletal gain from SOST antibody treatment (29). Herein, our immunofluorescence double staining demonstrated the interactions between SOST and LRP5/6 in UM cells, proving that SOST directly bound to LRP5/6 membrane receptors, thereby inhibiting Wnt/β-catenin signaling during UM progression.

To confirm the above demonstration, this study also used the human UM cell line OCM-1 to construct a stable SOST knockdown expression cell line. Thereafter, we established an animal model of ocular orthotopic tumor implantation in BALB/c nude mice to verify the effects of SOST inhibition on UM tumor formation. The results showed that when SOST expressions were knocked down in human UM cells, the tumor volume of ocular tumors of nude mice was larger. Additionally, the expressions of Wnt-1, β-catenin, Cyclin-D1, and MMP2 as well as MMP9 were all increased when the expression of SOST was suppressed in UM cells. As demonstrated by Kim et al. SOST is capable of inactivating Wnt signaling in Xenopus embryos (30). Furthermore, Zhou et al. further proved that the expression of cyclin-D1 is linked with the progression of UM, and high Let-7b expression may weaken the expression of cyclin-D1 and heighten cell cycle arrest to increase the radiosensitivity of UM cells (31). Hou et al. demonstrated that MMP2 is a downstream effector affecting UM metastasis, and miR-34a negatively modulates LGR4, reducing the expression of MMP2 to inhibit UM cell migration and invasion (32). Chang et al. verified that epigallocatechin gallate may downregulate the activity of secreted MMP-2 *via* suppression of ERK1/2 phosphorylation, and thus reduces the migration of UM cells (33). Cheng et al. demonstrated that HMGA1 can accelerate the progression of UM *via* positively modulating the activity of PI3K/Akt/MMP9 signaling in xenograft murine models (34). The above evidence demonstrated that SOST suppression may heighten UM progression through activating Wnt/β-catenin signaling *via* binding to membrane receptors LRP5/LRP6.

## Conclusion

Taken together, this research proposed that SOST suppression may activate the Wnt/β-catenin pathway, thereby accelerating UM malignant progression. We also have reasons to believe that the SOST-triggered Wnt/β-catenin pathway might represent a promising therapeutic as well as adjunctive regimen against UM. Despite this, future clinical trials are needed for determining the safety as well as effects of this therapy. Due to the small number of subjects, it is difficult to generalize the results. To gain a deeper understanding of the biological behaviors of UM cells as well as better assess the prognosis of patients, clinical trials and clinical studies on a larger population based on the SOST-triggered Wnt/β-catenin pathway are also needed.

## Data Availability Statement

The original contributions presented in the study are included in the article/supplementary material. Further inquiries can be directed to the corresponding authors.

## Ethics Statement

The studies involving human participants were reviewed and approved by Tianjin Medical University Eye Hospital (2017KY(L)-53). The patients/participants provided their written informed consent to participate in this study. The animal study was reviewed and approved by Tianjin Medical University Eye Hospital (TJYY2020122054).

## Author Contributions

TW and XH conceived and designed the study. HW and SZ conducted most of the experiments and data analysis and wrote the manuscript. YL and FS participated in collecting data and helped to draft the manuscript. All authors reviewed and approved the manuscript.

## Funding

This study was supported by grants from The Science& Technology Development Fund of Tian jin Education Commission for Higher Education (NO.2020KJ176).

## Conflict of Interest

The authors declare that the research was conducted in the absence of any commercial or financial relationships that could be construed as a potential conflict of interest.

## Publisher’s Note

All claims expressed in this article are solely those of the authors and do not necessarily represent those of their affiliated organizations, or those of the publisher, the editors and the reviewers. Any product that may be evaluated in this article, or claim that may be made by its manufacturer, is not guaranteed or endorsed by the publisher.

## Abbreviations

UM, uveal melanoma; SOST, sclerostin; HUVEC, human umbilical vein endothelial cells; DMEM, Dulbecco’s modified eagle medium; FBS, fetal bovine serum; si-NC, negative control siRNA; si-SOST, SOST siRNA; RT-qPCR, real-time quantitative polymerase chain reaction; GAPDH, glyceraldehyde phosphate dehydrogenase; MTT, methyl thiazolyl tetrazolium; sh-SOST, SOST shRNA; sh-NC, negative control shRNA; ANOVA, one-way analysis of variance.

## References

[1] Bustamante P Piquet L Landreville S Burnier JV. Uveal Melanoma Pathobiology: Metastasis to the Liver. Semin Cancer Biol (2021) 71:65–85. doi: 10.1016/j.semcancer.2020.05.003 32450140

[2] Beasley AB Chen FK Isaacs TW Gray ES. Future Perspectives of Uveal Melanoma Blood Based Biomarkers. Br J Cancer (2022) 126(11):1511–28. doi: 10.1038/s41416-022-01723-8 PMC9130512 35190695

[3] Ambrosini G Rai AJ Carvajal RD Schwartz GK. Uveal Melanoma Exosomes Induce a Prometastatic Microenvironment Through Macrophage Migration Inhibitory Factor. Mol Cancer Res (2022) 20(4):661–9. doi: 10.1158/1541-7786.Mcr-21-0526 34992145

[4] Medina CA Biscotti CV Singh N Singh AD. Diagnostic Cytologic Features of Uveal Melanoma. Ophthalmology (2015) 122(8):1580–4. doi: 10.1016/j.ophtha.2015.04.013 26012864

[5] Seibel I Cordini D Rehak M Hager A Riechardt AI Böker A. Local Recurrence After Primary Proton Beam Therapy in Uveal Melanoma: Risk Factors, Retreatment Approaches, and Outcome. Am J Ophthalmol (2015) 160(4):628–36. doi: 10.1016/j.ajo.2015.06.017 26133249

[6] Jang GF Crabb JS Hu B Willard B Kalirai H Singh AD. Proteomics of Primary Uveal Melanoma: Insights Into Metastasis and Protein Biomarkers. Cancers (Basel) (2021) 13(14):3520. doi: 10.3390/cancers13143520 34298739 PMC8307952

[7] Kim S Kim SA Nam GH Hong Y Kim GB Choi Y. *In Situ* Immunogenic Clearance Induced by a Combination of Photodynamic Therapy and Rho-Kinase Inhibition Sensitizes Immune Checkpoint Blockade Response to Elicit Systemic Antitumor Immunity Against Intraocular Melanoma and its Metastasis. J Immunother Cancer (2021) 9(1):e001481. doi: 10.1136/jitc-2020-001481 33479026 PMC7825261

[8] Pandiani C Strub T Nottet N Cheli Y Gambi G Bille K. Single-Cell RNA Sequencing Reveals Intratumoral Heterogeneity in Primary Uveal Melanomas and Identifies HES6 as a Driver of the Metastatic Disease. Cell Death Differ (2021) 28(6):1990–2000. doi: 10.1038/s41418-020-00730-7 33462406 PMC8185008

[9] Koch EAT Petzold A Wessely A Dippel E Gesierich A Gutzmer R. Immune Checkpoint Blockade for Metastatic Uveal Melanoma: Patterns of Response and Survival According to the Presence of Hepatic and Extrahepatic Metastasis. Cancers (Basel) (2021) 13(13):3359. doi: 10.3390/cancers13133359 34283061 PMC8268645

[10] Cherepakhin OS Argenyi ZB Moshiri AS. Genomic and Transcriptomic Underpinnings of Melanoma Genesis, Progression, and Metastasis. Cancers (Basel) (2021) 14(1):123. doi: 10.3390/cancers14010123 35008286 PMC8750021

[11] Ren R Du Y Niu X Zang R. ZFPM2-AS1 Transcriptionally Mediated by STAT1 Regulates Thyroid Cancer Cell Growth, Migration and Invasion *via* miR-515-5p/TUSC3. J Cancer (2021) 12(11):3393–406. doi: 10.7150/jca.51437 PMC8100800 33976749

[12] Zhao G Yin Y Zhao B. miR-140-5p Is Negatively Correlated With Proliferation, Invasion, and Tumorigenesis in Malignant Melanoma by Targeting SOX4 *via* the Wnt/β-Catenin and NF-κb Cascades. J Cell Physiol (2020) 235(3):2161–70. doi: 10.1002/jcp.29122 31385607

[13] Wu S Han M Zhang C. Overexpression of microRNA-130a Represses Uveal Melanoma Cell Migration and Invasion Through Inactivation of the Wnt/β-Catenin Signaling Pathway by Downregulating USP6. Cancer Gene Ther (2021). doi: 10.1038/s41417-021-00377-7 34522027

[14] Zheng L Pan J. The Anti-Malarial Drug Artesunate Blocks Wnt/β-Catenin Pathway and Inhibits Growth, Migration and Invasion of Uveal Melanoma Cells. Curr Cancer Drug Targets (2018) 18(10):988–98. doi: 10.2174/1568009618666180425142653 29692251

[15] Dean DB Watson JT Jin W Peters C Enders JT Chen A. Distinct Functionalities of Bone Morphogenetic Protein Antagonists During Fracture Healing in Mice. J Anat (2010) 216(5):625–30. doi: 10.1111/j.1469-7580.2010.01214.x PMC2871998 20298438

[16] Kim SP Da H Wang L Taketo MM Wan M Riddle RC. Bone-Derived Sclerostin and Wnt/β-Catenin Signaling Regulate Pdgfrα(+) Adipoprogenitor Cell Differentiation. FASEB J (2021) 35(11):e21957. doi: 10.1096/fj.202100691R 34606641 PMC8496915

[17] Wu T Wang LN Tang DR Sun FY. SOST Silencing Promotes Proliferation and Invasion and Reduces Apoptosis of Retinoblastoma Cells by Activating Wnt/β-Catenin Signaling Pathway. Gene Ther (2017) 24(7):399–407. doi: 10.1038/gt.2017.31 28485721

[18] Zou J Zhang W Li XL. Effects of SOST Gene Silencing on Proliferation, Apoptosis, Invasion, and Migration of Human Osteosarcoma Cells Through the Wnt/β-Catenin Signaling Pathway. Calcif Tissue Int (2017) 100(6):551–64. doi: 10.1007/s00223-016-0231-6 28246931

[19] Semënov M Tamai K He X. SOST is a Ligand for LRP5/LRP6 and a Wnt Signaling Inhibitor. J Biol Chem (2005) 280(29):26770–5. doi: 10.1074/jbc.M504308200 15908424

[20] Wang X Cheng G Miao Y Qiu F Bai L Gao Z. Piezo Type Mechanosensitive Ion Channel Component 1 Facilitates Gastric Cancer Omentum Metastasis. J Cell Mol Med (2021) 25(4):2238–53. doi: 10.1111/jcmm.16217 PMC7882944 33439514

[21] Pelster MS Gruschkus SK Bassett R Gombos DS Shephard M Posada L. Nivolumab and Ipilimumab in Metastatic Uveal Melanoma: Results From a Single-Arm Phase II Study. J Clin Oncol (2021) 39(6):599–607. doi: 10.1200/jco.20.00605 33125309 PMC8257877

[22] Nathan P Hassel JC Rutkowski P Baurain JF Butler MO Schlaak M. Overall Survival Benefit With Tebentafusp in Metastatic Uveal Melanoma. N Engl J Med (2021) 385(13):1196–206. doi: 10.1056/NEJMoa2103485 34551229

[23] Chang JC Christiansen BA Murugesh DK Sebastian A Hum NR Collette NM. SOST/Sclerostin Improves Posttraumatic Osteoarthritis and Inhibits MMP2/3 Expression After Injury. J Bone Miner Res (2018) 33(6):1105–13. doi: 10.1002/jbmr.3397 PMC6033030 29377313

[24] van Bezooijen RL ten Dijke P Papapoulos SE Löwik CW. SOST/sclerostin, an Osteocyte-Derived Negative Regulator of Bone Formation. Cytokine Growth Factor Rev (2005) 16(3):319–27. doi: 10.1016/j.cytogfr.2005.02.005 15869900

[25] Li J Xue J Jing Y Wang M Shu R Xu H. SOST Deficiency Aggravates Osteoarthritis in Mice by Promoting Sclerosis of Subchondral Bone. BioMed Res Int (2019) 2019:7623562. doi: 10.1155/2019/7623562 31828128 PMC6885161

[26] Hudson BD Hum NR Thomas CB Kohlgruber A Sebastian A Collette NM. SOST Inhibits Prostate Cancer Invasion. PloS One (2015) 10(11):e0142058. doi: 10.1371/journal.pone.0142058 26545120 PMC4636315

[27] Hesse E Schröder S Brandt D Pamperin J Saito H Taipaleenmäki H. Sclerostin Inhibition Alleviates Breast Cancer-Induced Bone Metastases and Muscle Weakness. JCI Insight (2019) 5(9):e125543. doi: 10.1172/jci.insight.125543 PMC6538320 30965315

[28] Ciubean AD Ungur RA Irsay L Ciortea VM Borda IM Dogaru GB. Polymorphisms of FDPS, LRP5, SOST and VKORC1 Genes and Their Relation With Osteoporosis in Postmenopausal Romanian Women. PloS One (2019) 14(11):e0225776. doi: 10.1371/journal.pone.0225776 31774873 PMC6880991

[29] Lim KE Bullock WA Horan DJ Williams BO Warman ML Robling AG. Co-Deletion of Lrp5 and Lrp6 in the Skeleton Severely Diminishes Bone Gain From Sclerostin Antibody Administration. Bone (2021) 143:115708. doi: 10.1016/j.bone.2020.115708 33164872 PMC7770084

[30] Kim J Han W Park T Kim EJ Bang I Lee HS. Sclerostin Inhibits Wnt Signaling Through Tandem Interaction With Two LRP6 Ectodomains. Nat Commun (2020) 11(1):5357. doi: 10.1038/s41467-020-19155-4 33097721 PMC7585440

[31] Zhou Y Zhang L Fan J Jia R Song X Xu X. Let-7b Overexpression Leads to Increased Radiosensitivity of Uveal Melanoma Cells. Melanoma Res (2015) 25(2):119–26. doi: 10.1097/cmr.0000000000000140 25588203

[32] Hou Q Han S Yang L Chen S Chen J Ma N. The Interplay of MicroRNA-34a, LGR4, EMT-Associated Factors, and MMP2 in Regulating Uveal Melanoma Cells. Invest Ophthalmol Vis Sci (2019) 60(13):4503–10. doi: 10.1167/iovs.18-26477 31661551

[33] Chang CW Hsieh YH Yang WE Yang SF Chen Y Hu DN. Epigallocatechingallate Inhibits Migration of Human Uveal Melanoma Cells *via* Downregulation of Matrix Metalloproteinase-2 Activity and ERK1/2 Pathway. BioMed Res Int (2014) 2014:141582. doi: 10.1155/2014/141582 25184134 PMC4145379

[34] Cheng Y Cheng T Zhao Y Qu Y. HMGA1 Exacerbates Tumor Progression by Activating miR-222 Through PI3K/Akt/MMP-9 Signaling Pathway in Uveal Melanoma. Cell Signal (2019) 63:109386. doi: 10.1016/j.cellsig.2019.109386 31394192

